# Supplementary figures and images for: Poxviruses package viral redox proteins in lateral bodies and modulate the host oxidative response
Source: PLoS Pathog. 2022 Jul 14;18(7):e1010614. doi: 10.1371/journal.ppat.1010614 (PMC9282662; doi:10.1371/journal.ppat.1010614)

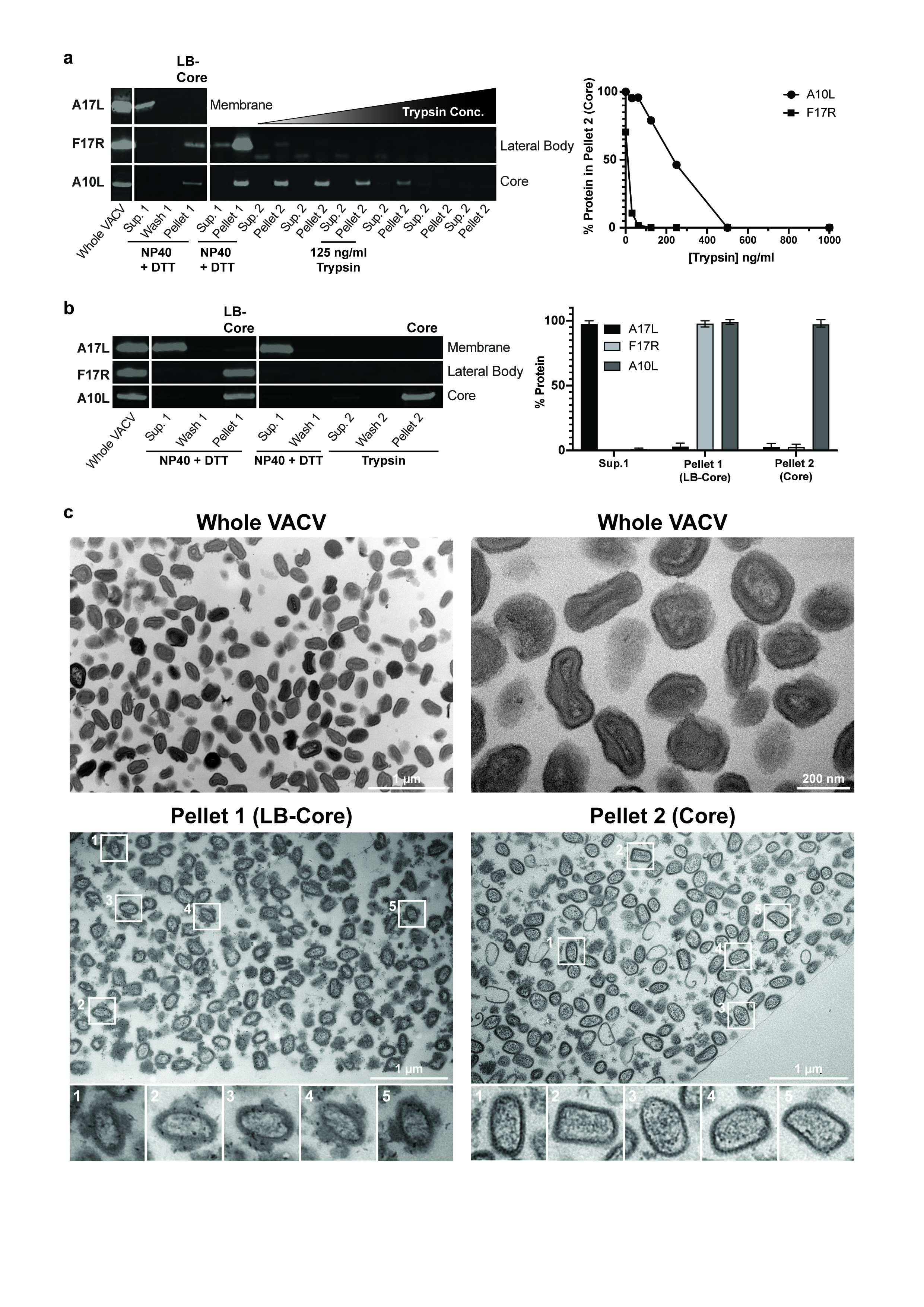

Supplement: S1 Fig — (a) Left: VACV MV fractionation and LB digestion conditions were optimised using WT VACV MVs. Virion membranes (Sup. 1) were removed by treatment with NP-40 + 50 mM DTT. To assess optimal conditions for LB removal, LB-Core samples (Pellet 1) were treated with various trypsin concentrations (31.25, 62.5, 125, 250, 500, 1000 ng/ml). The corresponding soluble (Sup. 2) and insoluble Core samples (Pellet 2) were retained. Viral fraction samples were analysed by immunoblotting against A17L (membrane protein), F17R (LB protein) and A10L (core protein). Right: the percentage of A10L and F17R residing in Pellet 2 (Core) upon increasing trypsin concentration was quantified using imageJ ‘analyse gels’ tool (see materials and methods). A single example image and quantification are displayed. A trypsin concentration of 125 ng/ml was selected, from multiple experiments, for preparing MS samples (b) Left: Representative immunoblot of A17L (membrane protein), F17R (LB protein) and A10L (core) from a large-scale fractionation performed, as determined in (a), for MS analysis. Right: Quantification of the percentage of A17L, F17R and A10L in Sup. 1 (Membrane), Pellet 1 (LB-Core) and Pellet 2 (Core) samples (n = 3; ± SEM). (c) WT purified VACV MVs (Whole VACV) were fractionated as in (a). Pellet 1 (LB-Core) and Pellet 2 (Core) samples were washed after the trypsin treatment, fixed, sectioned and imaged by TEM (Scale bars = 1 μm). (TIFF) [file ppat.1010614.s001.tiff]

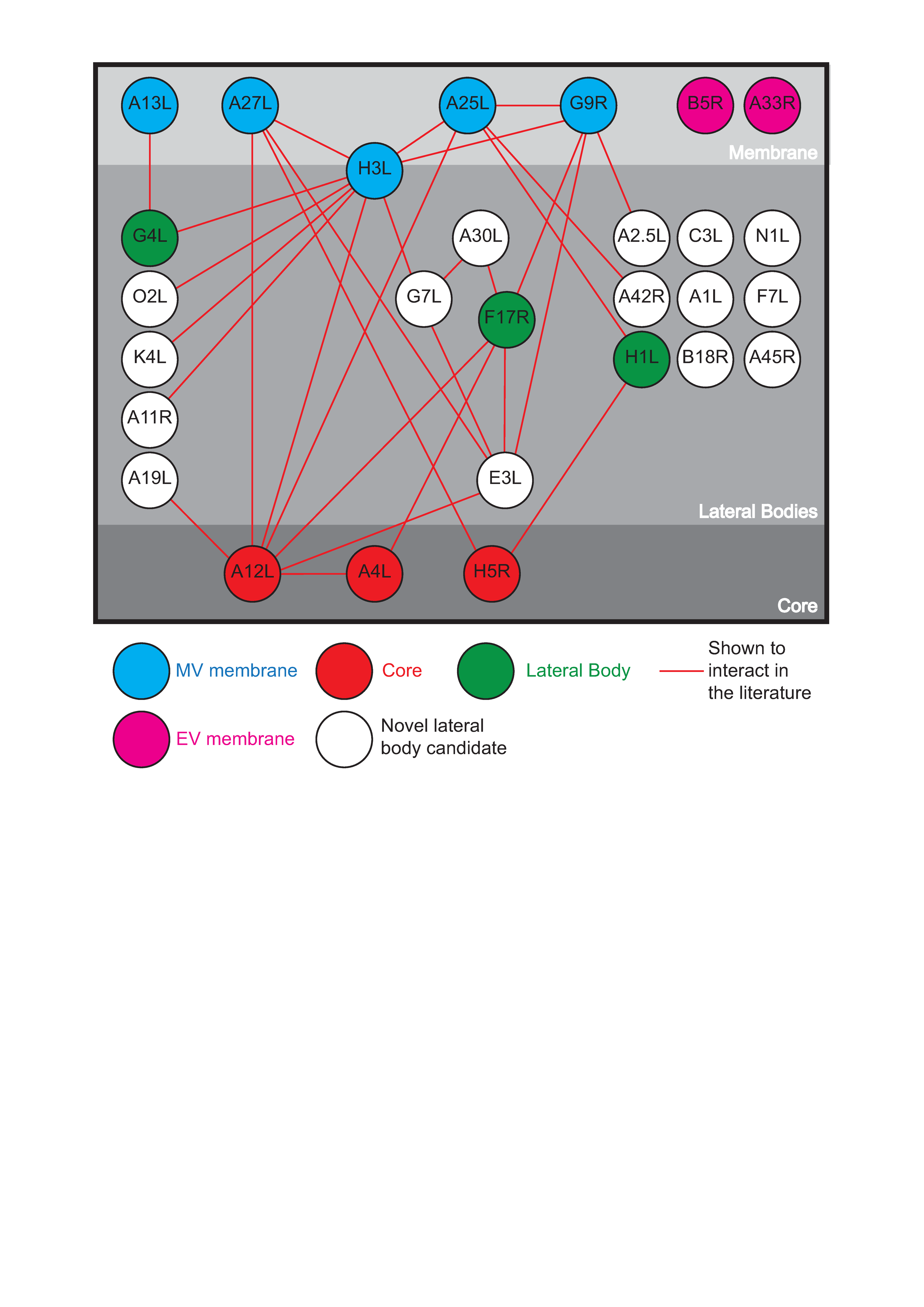

Supplement: S2 Fig — The VACV-MV protein-protein interaction network established by Mirzakhanyan and Gershon (65) was used to build a schematic of the interactions of the LB candidate proteins identified by this study. Proteins are colour-coded according to subviral location as indicated. (TIF) [file ppat.1010614.s002.tif]

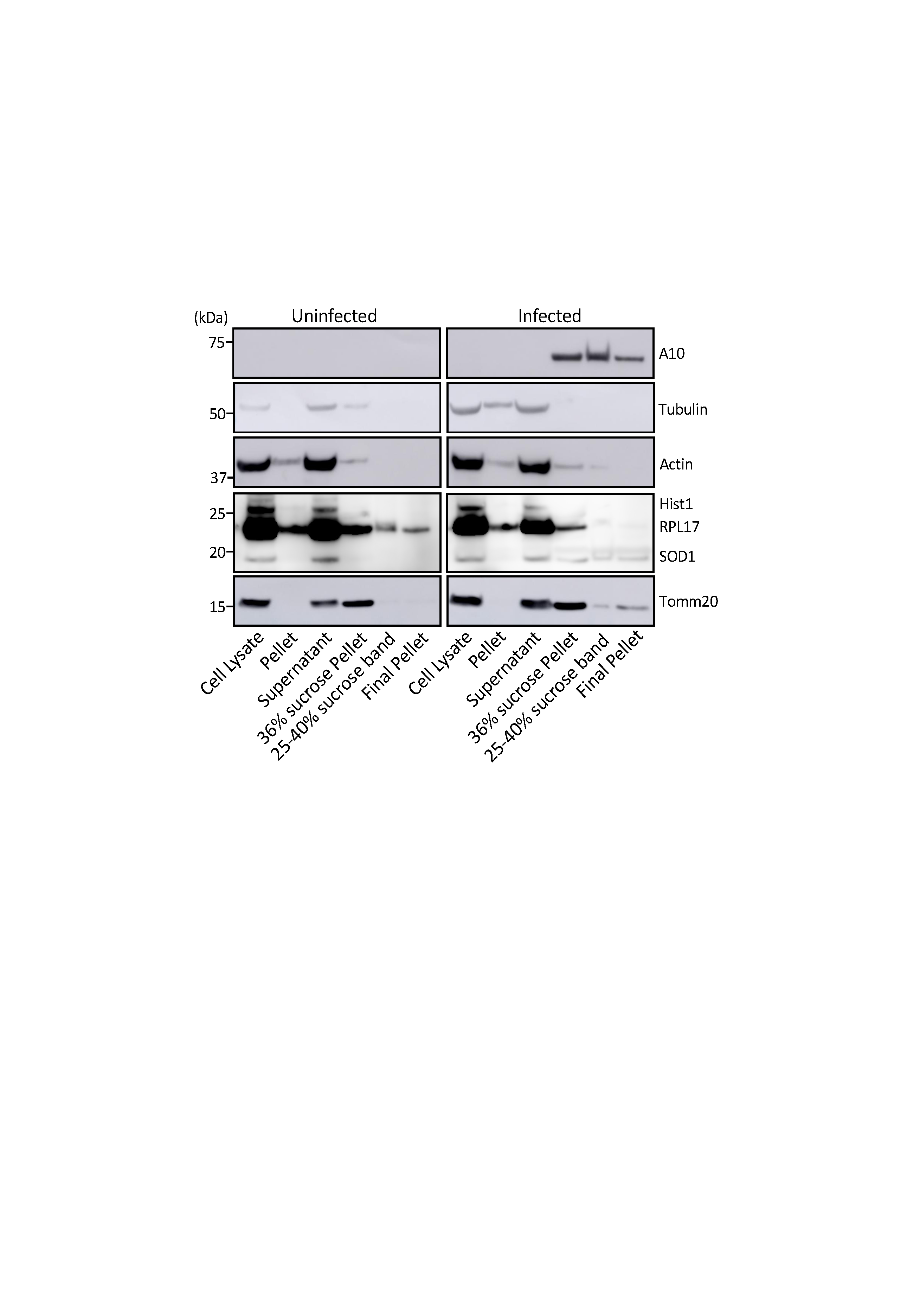

Supplement: S3 Fig — Immunoblot analysis of cellular proteins identified as not enriched (actin, tubulin) or enriched (Hist1, RPL17, SOD1 and Tomm20) in LBs by comparative quantitative MS (Fig 2 and S3 Table). The VACV purification protocol was performed on uninfected and infected cell lysates and samples collected for analysis at multiple stages [Post cell lysis (cell lysate/nuclear pellet), pellet from 36% sucrose cushion, virus band from 25–40% sucrose gradient, and final virus pellet]. Experiments were performed in duplicate and representative blots shown. (TIF) [file ppat.1010614.s003.tif]

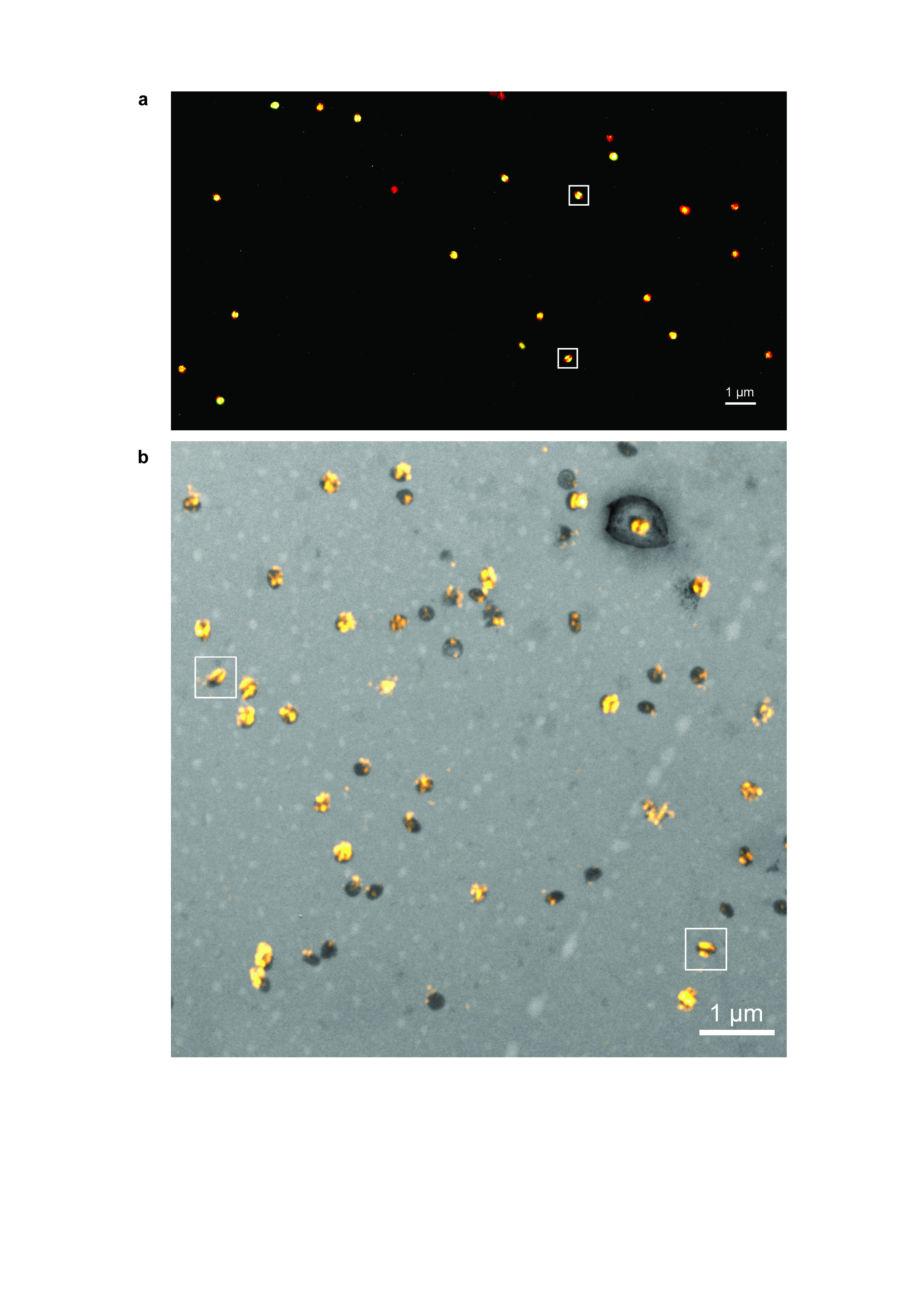

Supplement: S4 Fig — (a) Correlative SIM / STORM of WR mCherry-A4L A19L-EGFP virions. The two viral particles shown in Fig 3E are boxed. (b) Correlative STORM / EM of WR mCherry-A4L A19L-EGFP virions immunolabelled with anti-GFP nanobody. STORM images of the lateral body protein were registered with EM micrographs. The two viral particles shown in Fig 3F are boxed. Scale bars = 1 μm. (TIFF) [file ppat.1010614.s004.tiff]

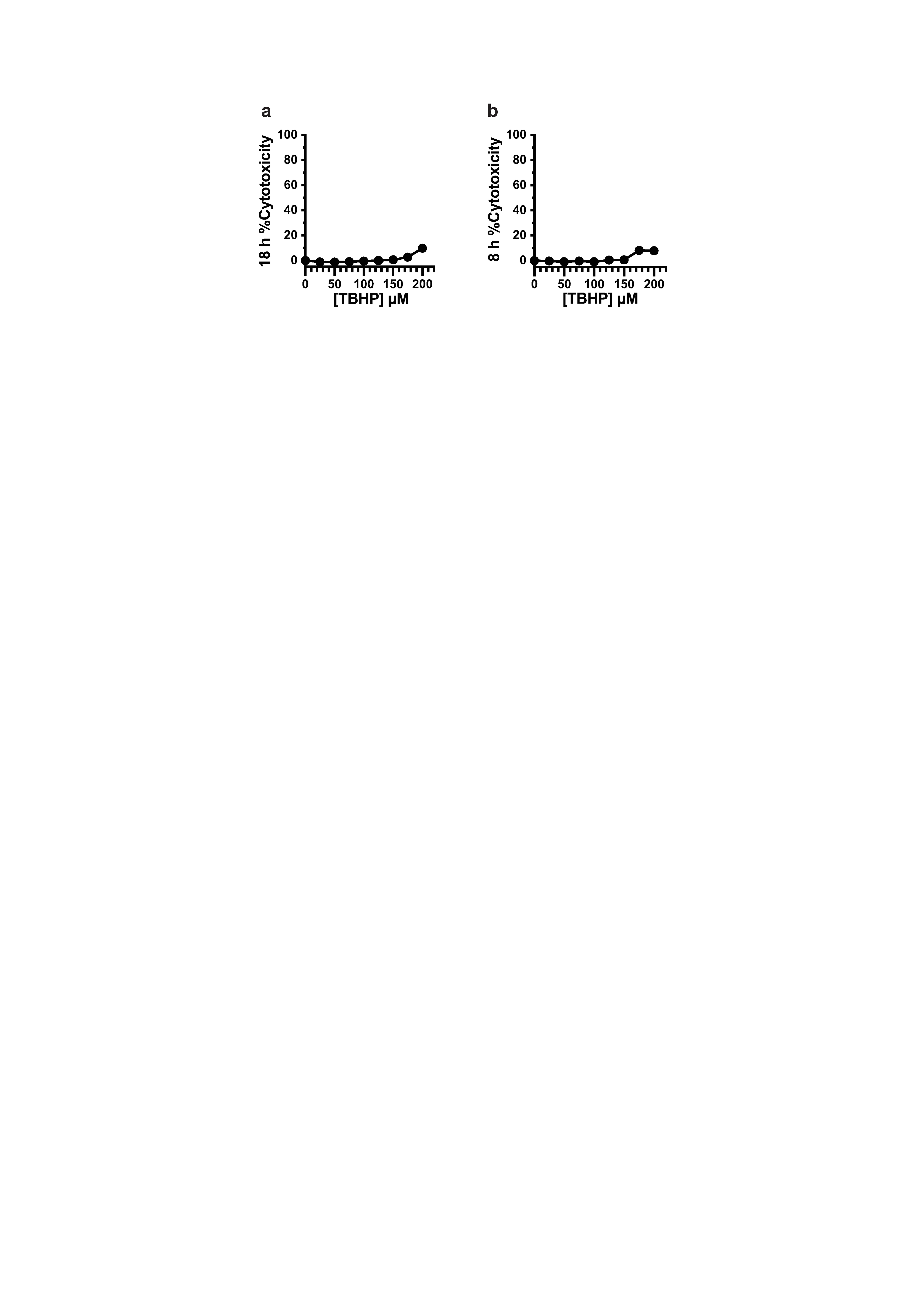

Supplement: S5 Fig — TBHP treatment is not cytotoxic (a) LDH release assay for cytotoxicity of TBHP titration on A549s cells under the conditions used in Fig 4C. (b) LDH release assay for cytotoxicity of TBHP titration on A549s cells under the conditions used in Fig 4D and 4E (n = 3 ± SEM). (TIF) [file ppat.1010614.s005.tif]
