# Supplementary material for: Poxviruses package viral redox proteins in lateral bodies and modulate the host oxidative response
Source: PLoS Pathog. 2022 Jul 14;18(7):e1010614. doi: 10.1371/journal.ppat.1010614 (PMC9282662; doi:10.1371/journal.ppat.1010614)
Supplement: Attachment — Submitted filename: 117444_1_rebuttal_2164712_qypd64.pdf [file ppat.1010614.s009.pdf]

Revision Plan

Review COMMONS

Manuscript number: RC-2021-00848
Corresponding author(s): Jason, Mercer

[The "revision plan" should delineate the revisions that authors intend to carry out in response to the points raised by the referees. It also provides the authors with the opportunity to explain their view of the paper and of the referee reports.

The document is important for the editors of affiliate journals when they make a first decision on the transferred manuscript. It will also be useful to readers of the reprint and help them to obtain a balanced view of the paper.

If you wish to submit a full revision, please use our "Full Revision" template. ***It is important to use the appropriate template to clearly inform the editors of your intentions.****]*

1. General Statements [optional]

We thank the reviewers for their comments and suggestions. We believe that many of the concerns arise from misunderstanding of the vaccinia virus (VACV) purification and fractionation procedures established and accepted by the poxvirus field. This implies that our introduction, description of procedures and explanation of the results needs clarification. We will update these sections of the manuscript to include more comprehensive introduction and description to provide a manuscript which is more accessible to non-specialist readers.

Having taken the reviewers on board, we also propose additional experiments as detailed below.

Description of the planned revisions
Insert here a point-by-point reply that explains what revisions, additional experimentations and analyses are planned to address the points raised by the referees.

Our responses to the reviewers' comments are in line below.

Reviewer #1 (Evidence, reproducibility and clarity (Required)):

Electron microscopic images of vaccinia virus (VACV) mature virions (MVs) reveal three main structures: an outer membrane, a core, and lateral bodies (LBs) between the core and membrane. It has been suggested that the components of the lateral body might interact with the host to prevent anti-viral defenses. Earlier studies from this laboratory used super resolution microscopy to localize three viral proteins to the LBs: a phosphoprotein, a phosphatase, and a glutaredoxin. The object of the first part of the paper was to identify additional proteins associated with the LBs. Having found some redox proteins, they investigated whether VACV modulates oxidative anti-viral responses upon infection.

Revision Plan

Major Comments

1.The study begins with the characterization of viral and cellular proteins associated with fractions derived from purified VACV MVs. However, the authors fail to provide evidence of purity of the virus particles isolated from cell lysates, nor is the purity described in the reference given for the method used. The electron micrographs of core-LB and core fractions (Fig. S1) show a large amount of apparently adventitious material. Many of the viral proteins associated with these fractions, such as those belonging to the extracellular enveloped form of the virus, and the 586 cell proteins of which 210 were associated with the core and core-lateral body fractions, particularly those associated with mitochondria, could be from contaminating cellular material rather than incorporated into virus particles. However, neither the purity of the particles nor this interpretation is discussed and the abstract and text refer to these cell proteins as if they were bona fide core and LB-associated proteins. For this interpretation to be credible, strong evidence for purity of the virus particles and subfractions need to be provided.

VACV virions were purified using the standard methodology established by the poxvirus field. This includes the combination of a sucrose cushion, followed by virion banding (isolation) on a 25-40% sucrose gradient. This procedure results in highly purified VACV virions free of cellular contaminants. As an example of the quality and purity of VACV virions isolated using this procedure please see supplementary Figure 3B. This VACV preparations only contain virions, there is no evidence of membranous cellular contamination (*i.e* mitochondria, golgi ER). In addition, numerous EM, cryoEM, and super-resolution imaging and mass spectrometry studies have utilized this purification procedure as below:

-Chung CS, Chen CH, Ho MY, Huang CY, Liao CL, Chang W. Vaccinia virus proteome: identification of proteins in vaccinia virus intracellular mature virion particles. J Virol. 2006;80(5):2127-40.

-Cyrklaff M *et al.* Cryo-electron tomography of vaccinia virus. PNAS. 2005; 102 (8) 2772-2777

-Gray RD, Beerli C, Pereira PM, Scherer KM, Samolej J, Bleck CK, et al. VirusMapper: open-source nanoscale mapping of viral architecture through super-resolution microscopy. Sci Rep. 2016;6:29132.

The electron micrographs shown in Figure S1 are of the virions after treatment with NP40/DTT and after treatment with NP40/DTT, followed by trypsin. The “debris” seen in these images are the viral components removed by this fractionation protocol, as was the intent of this experiment.

This is a comparative mass spectrometry study which only identifies proteins whose abundance has been changed by treatment with Trypsin to remove LB proteins. Critically prior to the trypsin treatment all samples have been purified through 36% sucrose, band purified on a 25-40% sucrose gradient, stripped of their outer viral membrane with NP40 + DTT treatment, pelleted by centrifugation to remove

solubilised material, washed and pelleted again. As a result, the proteins identified are strongly associated with viral cores or cores+LBs as they have been able to withstand these multiple purification steps. Taking the comments around mitochondrial contamination as an example as all of the samples have undergone stringent NP40/DTT treatment, the mitochondria would be lysed and their contents would reside in the soluble fraction which is washed away. Moreover, no mitochondria can be seen in the EM images shown in Fig S3B or Figure S1. Thus, we believe that the mitochondrial proteins detected as enriched in LBs are real candidates that require consideration and future study.

Many cellular proteins were identified as potentially residing in LBs. The subviral location of some of these cellular proteins has been studied by our research team. We recently confirmed the LB presence of Vimentin (Wood *et al.* Cell Micro 2021). However, confirming the LB location of host proteins identified here is out of the scope of this manuscript but rather the subject of future publications.

To address the reviewers concerns we complete this figure, we will add EM images of the purified VACV virions prior to treatment which illustrate a lack of cellular contamination, and we will add additional explanation and discussion of the fractionation procedure and its results to the abstract, introduction results and discussion sections of the manuscript.

2.The identification of LB proteins depends on the removal of the membrane with a detergent, pelleting of the particulate material which is referred to as the core-LB fraction, followed by trypsin digestion and again pelleting the particulate material which is referred to as the core fraction. While trypsin digestion removes the lateral bodies, it does not mean that all proteins removed by trypsin are lateral body proteins. Therefore, the purification method is an enrichment and candidate LB proteins must be verified by other procedures, which was only done for 5 proteins

This is correct. The trypsin treatment enables the identification through comparative enrichment of both LB proteins and proteins associated with the outer surface of the viral core. As a result, proteins that were already known to be bone-fide outer core and membrane proteins were discounted from the list of suggested LB candidate proteins.

Five proteins were confirmed to reside in the LBs by super-resolution microscopy. Other candidate proteins were confirmed by super-resolution imaging but not shown in the manuscript as they were out of the scope of the manuscript's focus and are the subject of future studies. We would like to stress that for each candidate LB protein that we visualize by super-resolution imaging, a recombinant virus expressing a fluorescent-tagged version of the candidate protein must be generated. The construction of recombinant viruses takes a significant amount of time. In addition, inner components of VACV virions cannot be visualized using antibodies, as they do not penetrate the highly proteinaceous VACV membrane even after NP-40 treatment. As such we chose to focus on the 5 redox proteins shown in this manuscript as they are the ongoing focus of the study and are exemplary that the mass spectrometry approach had identified bone-fide lateral body proteins.

In addition, the full list of potential LB proteins has been provided as a resource for the field. We encourage researchers to confirm the sub-viral location of proteins if that is key to their research story.

3.The abstract states that 15 viral LB proteins were identified. However, only five of the 15 were further analyzed by super resolution microscopy in this study so that the others should be referred to as candidate LB proteins. Similarly, the cell proteins should be referred to as candidate LB proteins as none of them were analyzed by super resolution microscopy even though 6 of them were reported to be more abundant than the most abundant viral protein.

We can adjust the text to accommodate this comment. We propose to analyse the top 3-5 human proteins enriched in LBs using biochemical fractionation and immunoblotting. As explained above, we cannot visualize inner-virion proteins using antibodies thus super-resolution imaging of cellular LB components is not possible.

4.Fig. 4A shows a 3-fold reduction in ROS positive cells at 1 h after infection and much greater 23-fold reduction at 8 h. The higher inhibition at 8 h is attributed to post-replicative expression of LB proteins. However, by 8 h after infection, more than 100 viral proteins are expressed so why is this correlated with the LB proteins?

A small but significant impact of ROS is seen early during infection prior to viral gene expression. This indicates that the effect is mediated by a protein delivered by incoming viruses, in line with LB proteins. As there are 5 redox proteins residing in the LBs it is likely that this impact is due to these proteins' activity. By 8 hours post infection, these proteins have been newly expressed by the infecting viruses in far greater amounts than what is delivered by incoming virions.

That we see a minor effect upon their delivery and a much larger effect upon their expression is consistent with these proteins acting as LB-based ROS effectors.

We will clarify this in the text.

Minor comments

The last paragraph of Discussion and Abstract states that that all VACV-encoded redox proteins are in LBs. Are there other core or membrane proteins that have Cxx(x)C motifs? At least one, E10, a bona fide redox protein was not shown to be in LBs

Thank you for pointing this out. This manuscript and our previous findings (Schmidt *et al.* Cell Reports 2013) indicate that E10 does not reside in the LBs. We will alter the wording of the manuscript to reflect this.

Revision Plan

Reviewer #1 (Significance (Required)):

The most significant contribution of this study is evidence for the localization of four viral protein to LBs bringing the total number to 7. Additional candidate LB proteins were also suggested but not yet validated. While nearly 100 cellular proteins, some more abundant than the viral proteins, were also suggested to be LB proteins, contamination particularly with mitochondria was not ruled out. Interestingly, two of the LB proteins (A2.4 and G4) were known to be involved in the formation of disulfide bonds and additional ones have Cxx(x)C motifs suggesting that they may also be involved in redox. A small reduction in ROS positive cells was shown at 1 h after infection and a much larger reduction at 8 h; however, no evidence was presented to support the hypothesis that these reductions were mediated by LB proteins.

As outline above, we have proposed multiple text changes and additional experiments to adequately address all the points raised by the reviewer.

Reviewer #2 (Evidence, reproducibility and clarity (Required)):

Bidgood and colleagues have use quantitative mass spectrometry to characterise the composition of VACV lateral bodies, proteinaceous structures analogous to the tegument of herpesviruses that have enigmatic composition and functions but are thought to deliver a bolus of protein that can modulate the host-cell environment before viral gene expression. They identify that VACV LBs contain a number of redox-active viral proteins plus many host-derived proteins. They go on to show that VACV infection reduces the abundance of reactive oxygen species in infected cells and that induction of oxidative stress is detrimental to virus replication. This work should serve as a useful reference for the poxvirus field, and adds to the body of literature confirming that redox state is yet another important host-cell environmental factor that viruses, and in particular large DNA viruses, seek to control during infection.

Overall the paper is well written, the figures are clear, the experiments seem to have been performed in a technically robust manner and the description of the methods is sufficient to allow reproduction of their study.

There are a few major questions that arise:

1.While VACV replication is clearly more robust when the cell does not face oxidative stress (Fig. 4C), the redox capacity of LB-delivered redox-active proteins seems low (compare bottom panels of Fig. 4B). Do the authors think that there is a spatial aspect to the redox modulation by VACV LB-delivered proteins? This could be tested via microscopy by infecting cells expressing genetically-encoded redox sensitive reporters like Grx1-roGFP2.

This is an interesting point, and we have considered that the redox modulation by VACV is likely to be primarily cytoplasmic. We have suspected that the low redox capacity observed may be due to our choice of cyto/nuclear ROS reporter.

We will perform the experiment proposed by the reviewer to investigate the spatial aspect of ROS modulation by microscopy.

2.What is the relevance of the cellular proteins that co-fractionate with VACV LBs/cores? Many host-cell proteins or complexes are identified but the authors don't perform experiments to validate the presence of any of these proteins in LBs (e.g. using microscopy) and their (potential) functional relevance isn't addressed. The presence of so many mitochondrial and ribosomal proteins is particularly striking and raises concerns that mitochondria and ribosomes/polysomes may have co-sedimented with the VACV particles in the sucrose cushion experiment. If the authors have performed control experiments to discount this hypothesis they should be presented and, if not, this potential confounding factor should be noted.

The role of cellular proteins that co-fractionate with viral LB proteins in the viral replication cycle is an open question which this manuscript prompts. It demands further study and we hope will be of considerable interest to the poxvirology field. It is not possible that the mitochondrial and ribosome proteins have simply co-sedimented with the VACV MVs during sucrose purification. As this is a comparative mass spectrometry study, it only identifies proteins whose abundance has been changed by treatment with Trypsin to remove LB proteins. Prior to the trypsin treatment all samples have been stripped of their outer viral membrane with NP40 + DTT treatment, pelleted by centrifugation to remove solubilised material and washed with buffer. As a result, the cellular proteins identified are strongly associated with viral cores or cores+LBs, as they have been able to withstand these multiple purification steps. Significantly, mitochondria would be lysed by the NP40 +DTT treatment and their contents solubilised so there is little possibility that the presence of these cellular proteins enriched in LBs is simply an artefact of the sample preparation methodology. Thus, we believe that the mitochondrial proteins detected as enriched in LBs are real candidates that require consideration and further study.

We can adjust the text to accommodate this comment. We propose to analyse the top 3-5 human proteins enriched in LBs using biochemical fractionation and immunoblotting. As explained above in response to reviewer 1 point 2, we cannot visualize inner-virion proteins using antibodies thus super-resolution imaging of cellular LB components is not possible.

3.How relevant is the measurement of redox sensitivity in cultured cells to the context of VACV infection in vivo? Partial pressures of oxygen are higher in cell culture incubators than in animal issues, although rapid replication of immortalised cultured cells can lead to dramatic lowering of pericellular oxygen tension over the course of hours due to limited diffusion of O2 through culture medium. The authors should comment on the difference in oxidative stress experienced by cultured cells versus animal tissues

in light of their results.

We thank the reviewer for suggesting this, while the impact of ROS on viral infection is studied in tissue culture as a model system, it has been shown that NOS2 -/- mice are highly suspectable to ectromelia virus (mousepox) infection relative to WT mice. We will add this manuscript and discuss it implications in the context of our findings comment on the likely relevance of our findings *in vivo* in the updated version of the manuscript.

4.The authors do not explain how VACV membrane proteins A26L and A6L are contained within VACV cores. Are these truncated soluble forms of the protein, for example lacking the transmembrane regions, or are they likely to associate tightly with the cores at the poles of the virion such that they remain associated after detergent removal of the membrane? The authors should comment.

We were not surprised to find MV membrane proteins that remained with Core/LB fraction after fractionation, due to the complex highly interconnected nature of VACV particles. Several manuscripts dedicated to investigating the molecular interactions within VACV have shown interactions between membrane, core and LB proteins. We will add a more thorough discussion of our findings in the context of intra-virion interactions.

5.The authors state that F17R constitutes 69% of LB mass (page 9 paragraph 2) but also that 6 human proteins were enriched and "at a greater abundance than the major LB viral protein F17R". F17R can't constitute more than 50% of the LB mass if there are other proteins more abundant in the LBs - can the authors please explain this discrepancy?

We thank the author for pointing this out. This was a gross estimate that arose from a volumetric estimate of VACV LBs based on EM and AFM images, the copy number of F17 in virions and the specific partial volume of globular proteins (Schmidt et.al Cell reports, 2013). Any one or all 3 of the cellular proteins could be more abundant, but take up less space in the LB based on their specific partial volume.

None the less, we agree that the wording of this sentence is confusing and will remove this from the manuscript.

Minor comments:

1.The authors state that they show hierarchical clustering of the proteomic results (Figs 1C, 2C) but the dendrograms of the clustering are not shown. These should be included.

The dendrograms were removed for clarity. We will put them back into the figure in an updated version of the manuscript.

2.In supplementary figure 1A the authors show quantitation of the lateral body versus core protein composition following variable trypsin treatment. However, they don't describe how this quantitation was performed, nor how many times it was attempted.

The method used to treat the samples with variable trypsin concentrations is described in the Materials and methods under “Viral Fractionation”. The method used for quantification is described in the Materials and methods Immunoblotting. This assay in supplementary figure 1a was only performed once as this is a standard field assay and was used to illustrate what the fractions look like upon the various treatments. The data in supplementary figure 1b was performed in triplicate and is an accurate representation of how successful the viral fraction protocol used in the study separates viral membrane, LB and core components.

3.As described above for the viral cores, the authors should expressly comment on how they envisage that membrane proteins are identified in the LB proteome.

We will be sure to comment of this in an updated version of the manuscript. Once again, this finding could indicate that certain membrane proteins are associating with the internal viral structures.

Reviewer #2 (Significance (Required)):

This manuscript provides novel information in the form of a proteomic catalogue of viral proteins incorporated into VACV LBs, plus cellular proteins that may be potentially incorporated. It provides further evidence that LBs are functional protein-delivery structures that modulate the infected-cell environment before viral gene expression. It also complements literature from other viruses on the importance of modulating oxidative stress for productive virus infection.

This work will be of interest primarily to people studying poxvirus biology.

I am an expert in virus:host interactions with experience in proteomic analysis of virus-infected cells.

Reviewer #3 (Evidence, reproducibility and clarity (Required)):

The authors described the vaccinia virus lateral body (LB) proteome using quantitative comparative mass spectrometry. The majority of host proteins found were associated with mitochondria. In addition, 15 viral LB proteins were identified. Of the 15 viral LB proteins found, five are VACV redox proteins and their LB association was confirmed by state of the art microscopy (SIM-STORM and STORM-TEM). Finally, the authors demonstrate that vaccinia virus suppresses host cell oxidative responses, which may antagonize host antiviral responses. The data is presented in clear and comprehensive manner. Largely

the conclusions are supported by the data. Appropriate numbers of experimental replicates have been performed.

Major comments:

1-Most conclusions are convincing. However, the link between the identified redox proteins and the anti-oxidative effect of VACV is not entirely clear. Could the authors express single VACV proteins and/or active site mutants of the viral redox modifiers to strengthen their conclusion that these LB proteins are causative of the observed redox status changes?

We thank the reviewer for this suggestion. In association with this manuscript, we attempted to look at the effects of deleting single VACV redox proteins on cellular redox status. Unfortunately, we saw no impact. We suspect, as is commonly seen with VACV immunomodulation, that the packaging of 5 redox proteins serves for functional redundancy. As two of the redox proteins, A2.5 and G4, as essential for viral assembly we could not test these in a deletion background. In short, generating multi-deletion viruses which include essential proteins has hampered our progress on linking the VACV redox proteins to the observed cellular redox changes.

We appreciate the reviewer's suggestion and will attempt to express the individual VACV redox proteins in the absence of infection and investigate changes in cell redox state. We will also attempt to deplete the 5 redox proteins using siRNA to investigate the link between VACV redox proteins and cellular redox state during infection.

2-The statement that poxvirus redox proteins are modulators of host oxidative anti-viral responses is not (yet) fully supported by the data presented. This should be addressed experimentally or alternatively the claim should be removed from the abstract, which would make the study a rather descriptive, but nonetheless informative, relevant and methodologically well executed study.

We will perform the experiments suggested above.

3-The statement that poxvirus redox proteins are modulators of host oxidative anti-viral responses is not (yet) supported by the data presented. If deletion mutant viruses cannot be generated, possibly expression of the single viral redox proteins could strengthen the claim. Recombinant protein expression and measurement of redox status of the cells seems feasible.

As above we will attempt to express the individual VACV redox proteins in the absence of infection and investigate changes in cell redox state. We will also attempt to deplete the 5 redox proteins using siRNA to investigate the link between VACV redox proteins and cellualr redox state during infection.

4-Data and the methods are presented in such a way that they can be reproduced.

5-Experiments are largely adequately replicated and statistical analysis adequate. Statistical analysis for Fig. 4a is missing.
Statistical Analysis will be included for 4a in an updated version of the manuscript.

Proteomic analysis is performed according to current standards. Data deposition of the full proteomics dataset in a public database is highly recommended.
Mass spectrometry data will be deposited in a public database.

Minor comments:

•Prior studies are referenced appropriately.

•Text and figures clear and accurate except for minor suggestions below.

Fig. 1C/D; Fig 2A/B. Protein names displayed in the heat maps and Volcano plots are almost illegible.
The font size on the figures will be increased.

Fig. 1C; Fig 2A. A hierarchical clustering may be preferable to the heat map.
The dendrograms were removed for clarity in the figure. We will replace them in the revised manuscript.

Fig 4. Statistical analysis should be performed to demonstrate that ROS production is significantly reduced by VACV.
Statistical Analysis will be included for 4a in an updated version of the manuscript.

Reviewer #3 (Significance (Required)):
The authors describe the first proteomic characterisation of poxvirus LBs, leading to the identification of four novel VACV LB proteins. Furthermore, VACV is shown to suppress cellular oxidative responses, which coincided with increased infection levels. This work builds on previous publications (Schmidt et al, 2013, Liu et al, 2014) and further characterises VACV LBs. Furthermore, the additional anti-oxidative role of the encased proteins is proposed. Virologists, especially those studying poxviruses and host-pathogen interactions should be interested in and influenced by the reported findings.
Field of expertise: Virology, proteomics.

Revision Plan

Reviewer #4 (Evidence, reproducibility and clarity (Required)):

Summary:

The work presented by Bidgood et al from the Mercer lab extends their prior studies of the poxvirus lateral bodies. In recent years, there has been an emerging hypothesis that these unusual virion features may serve a function akin to the herpesvirus tegument, delivering proteins upon infection that modukate host processes. Here, they successfully optimize a protocol to prepare subviral "cores" and "cores + lateral bodies" and use mass spectrometry to identify and compare the proteomes of each preparation. Superresolution microscopy is used to validate some of the spatial assignments. The most striking finding is the lateral body localization of the group of viral proteins known to function in the redox pathway and form disulfide-bonds within cytoplasmic viral proteins. They present further analyses suggesting that vaccinia "actively" suppresses oxidative stress (10%, rather than 40% of cells are ROS+), and that treatments that induce oxidative estress (TBHP) have a modest impact on levels of early and late transcripts and on viral yield.

The approaches are innovative and validated, the data are interesting and open up a new direction for research, and the paper is well written. However, there are some things that need addressed:

Comments:

1)Can the authors comment on the completeness of the mass spec analysis of the cores? L4, which is a major core component, isn't listed on the heat map/ H5, another abunndant protein, is listed - but it's hard to tell from the heat map why its abundance doesn't seem to change between the core and core + lateral bodies and doesn't seem to be abundant anywhere. Are other known core proteins missing?

We have not done an extensive cross-comparison of our data set with known core components, we can do this and comment on this in the discussion of the revised manuscript. We purposefully only labeled proteins on the heat map that changed in the comparative analysis. While L4 was detected in the mass spectrometry (table S1 tab2, VP8) it was not "enriched" in cores. For both L4 and H5 we suspect this has to do with the nature of DNA binding/associated protein. As is common in mass spectrometry screens, not all proteins are degraded into peptides that fly well upon ionisation. This possibility for L4 is supported by major difficulties in detecting and labeling L4 in detergent and protease treated virions (Moussatche and Condit, Virology 2015). We can comment on this in the discussion in conjunction with the proposed core protein analysis.

2)In their study of the redox proteins, they stress that the induction of ROS by TBHP decreases the level of early and late mRNAs as well as reducing viral yiield. However, even 100 uM TBHP decreases viral yield ~2-fold, and transcript levels less than that. These effects seem MUCH more moderate/minimal than the text suggests. The authors should adjust the text to be more in line with the data. Given the

data, is it likely that this is why the redox proteins evolved and are delivered in the lateral bodies? Is there another possibility, or a more vulnerable cell type?

We will adjust the text to be more moderate and reflective of the data. The effect is small but significant. We hypothesize that the moderate phenotype seen with TBHP is due to the fact that VACV is bringing 5 redox proteins into the cell with the express purpose of shutting down the ROS response (i.e. it takes a lot of ROS production to overcome VACVs redox proteins). We have proposed experiments to address this in the response to reviewer 3 point 1. These include analysis of the impact of VACV redox proteins on cell redox state during exogenous expression of VACV redox proteins outside of the context of infection, and upon siRNA depletion of VACV redox proteins during infection.

We tried multiple cell lines, each of which gave similar results to those presented in the manuscript.

3)In describing the data in FIg 4 d-e, the authors conclude that the impact of TBHP is at the level of transcription, because transcript levels are moderately decreased. This conclusion is not justified - the impact could be at the level of transcript stability.

The impact could be at the level of transcription or transcript stability. The text will be clarified to reflect this.

4)In their introduction, the authors state conclusively that vaccinia virus enters cells by macropinocytosis. Although there is good evidence for this mode of entry (from the senior author of this work during an earlier career stage), there are other investigators who have reported that entry can also occur via direct fusion at the plasma membrane. The authors should modify their statement.

The authors will modify their statement and include the associated references.

Reviewer #4 (Significance (Required)):

The work is well done and significant, and the authors have advance dour understanding of poxviral lateral bodies. The combination of different technical approaches is superb.

The encapsidation of the viral redox proteins into the cytoplasm upon entry is intriguing. Despite the rather modest impact that the authors show upon inducing oxidative stress, the hypothesis that vaccinia actively combats ROS immediately upon infection is provocative and worth studying.

The work will be of interest to a broad audience of virologists.

Revision Plan

Review COMMONS

2. Description of the revisions that have already been incorporated in the transferred manuscript

Please insert a point-by-point reply describing the revisions that were already carried out and included in the transferred manuscript. If no revisions have been carried out yet, please leave this section empty.

3. Description of analyses that authors prefer not to carry out

Please include a point-by-point response explaining why some of the requested data or additional analyses might not be necessary or cannot be provided within the scope of a revision. This can be due to time or resource limitations or in case of disagreement about the necessity of such additional data given the scope of the study. Please leave empty if not applicable.
