# Supplementary material for: Poxviruses package viral redox proteins in lateral bodies and modulate the host oxidative response
Source: PLoS Pathog. 2022 Jul 14;18(7):e1010614. doi: 10.1371/journal.ppat.1010614 (PMC9282662; doi:10.1371/journal.ppat.1010614)
Supplement: Attachment — Submitted filename: 00_Bidgood_Response_2_Reviewers.pdf [file ppat.1010614.s011.pdf]

Dear Derek, we thank you again for the opportunity to address the remaining reviewer concerns. As suggested, we have focused on expressing and assessing the redox potential of individual VACV LB proteins, and localization of a subset of cellular LB candidates to LBs as outlined in the point-by-point response below.

1) The authors could address the question of redundancy by expressing the candidate proteins in uninfected cells and treating with TBHP.

We first attempted to express the individual redox proteins as HA-tagged versions by cloning out the various genes from the viral genome and placing them in a mammalian expression vector under the control of a CMV promoter. While sequencing confirmed that the plasmids were correct, we did not observe expression of any of the proteins over a time course of 48 hours (not shown).

As we have previously had issues expressing virus proteins - cloned directly from the viral genome - in uninfected cells, we then had human codon optimized HA-tagged versions produced. Using these plasmids only O2 was expressed at a reasonable amount (left panel). We then tested its ability to suppress a TBHP SOX burst relative to a control plasmid and saw no impact (right panel). We include this data here for the reviewers, but without the full panel of proteins and co-expression experiments this adds little information to our study as is.

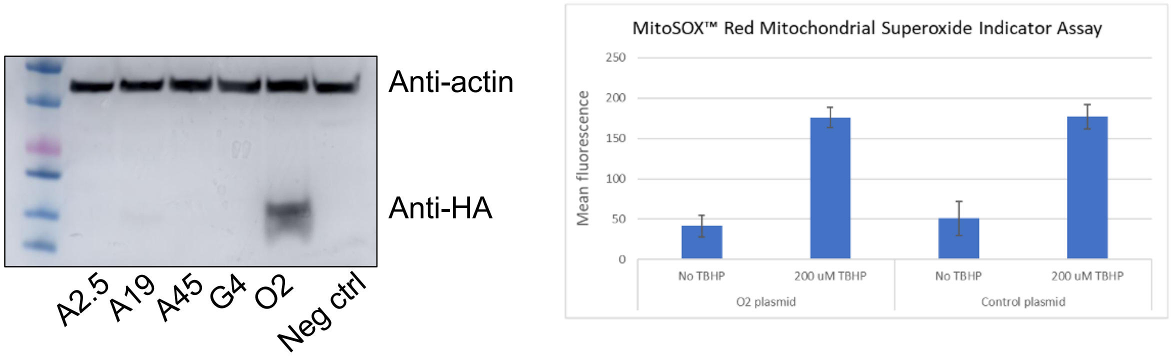

2) The authors could address the question of host cell LB proteins by using antibody and super resolution microscopy as they did for viral proteins.

We have now added dual-color SIM images of RPL17 and Tomm20 to the manuscript. (Please see Figure 3b). We attempted to permeabilize virions with saponin, but this was unsuccessful. To get antibodies to LBs we had to remove the membrane using our fractionation protocol. SIM imaging showed that both RPL17 and Tomm20 localize to LBs (visualized in both sagittal and frontal orientations.) Details are outlined below in the response to reviewer 4 and in the revised manuscript.

Reviewer #1: This article is well presented and the experiments are generally of high quality. The associated mass spectrometry data should prove a useful resource for the poxvirus field. The contribution of VACV redox proteins to virus replication has not been definitively demonstrated, but this study is likely to stimulate additional studies into the role of redox regulation in virus replication and the host-antiviral response.

We thank the reviewer for their appreciating of the work.

Reviewer #2: All comments and raised concerns have been adequately addressed.

Reviewer #3: The major strength of the paper is identifying a few additional viral proteins that localize in LBs. The weakness of the paper is that the other conclusions regarding host proteins localized in LBs and a role for LB proteins in combatting the host oxidative response are not convincing.

We have attempted to address both points through additional experiments as outlined below.

Reviewer #4: The manuscript is a very interesting piece of work which addresses the protein composition of the two lateral bodies (LBs) that are striking features of vaccinia virions. Virions are delimited by a membrane and contain a discrete core which has a proteinaceous wall and encloses the genome and a repertoire of transcriptional mediators. The lateral bodies flank the core, and there is an emerging sense that they contain bioactive, signaling molecules that set up a cytoplasmic milieu that supports vaccinia infection. Using a combination of virion fractionation, proteomics, and high-resolution microscopy, the authors document and discuss the contents of the lateral bodies.

In this revised manuscript, the authors have addressed many of the critiques raised after the first submission, and the work is significantly improved. They highlight two groups of proteins in particular: cellular proteins and virally encoded proteins that are known to be redox regulators and cellular proteins, many of which are normally associated with mitochondria.

The work is of high quality and the discussion is thorough. Undoubtedly, future studies will provide a more rigorous test of which of these "candidate lateral body" proteins are truly important for infection and are truly enriched in lateral bodies - but the current work is an important contribution to the field. To combat the possibility that cellular components may merely contaminate virions and LBs, they perform a "mock" virion purification from uninfected cells. Although imperfect because of the lack of the "mass" of sticky virion components, it is a good effort and a good addition. At some level, the concerns about impurities are the "nature of the beast". Localizing the cellular proteins to the LBs by high-resolution SIM/STORM would be a good addition, and is well within the expertise of these authors.

We thank the review for this assessment, their appreciation of the advancement this study brings to the field and their knowledge of the limitations of this system.

Part II – Major Issues: Key Experiments Required for Acceptance
Please use this section to detail the key new experiments or modifications of existing experiments that should be absolutely required to validate study conclusions.

Generally, there should be no more than 3 such required experiments or major modifications for a "Major Revision" recommendation. If more than 3 experiments are necessary to validate the study conclusions, then you are encouraged to recommend "Reject".

Reviewer #1: I have problems with interpretation of Figure S3, which is new data included in this revised manuscript. This figure supports that SOD1 and TOMM20 are, to some extent, found in the purified virus fraction (final pellet). Hist1 is not detected; this could reflect the differential sensitivity afforded by immunoblots versus mass spectrometry and its absence therefore neither supports nor refutes its specific association with virus particles. However, RLP17 is found at much higher abundance in the purified virus fraction (Final Pellet) in the uninfected cells than in the infected cells. To my eye it RLP17 is not at all visible in the infected cell sample (in contradiction to the statement on line 246-7). The authors don't seem to have critically assessed the data presented in Fig S3. This data shows that ribosomal proteins (presumably in the context of intact ribosomes) can indeed be purified when following the procedure used to isolate virus particle. This should be mentioned explicitly, as it suggests that identification of any ribosomal protein in the virion fraction should be interpreted with caution.

We thank the reviewer for this observation. We do see a minor RPL17 band in Fig. S3 and indicate in the manuscript that it is seen "to reduced amounts". Fig. 3a (virion fractionation data) shows more definitively that RPL17 is in virions and associated with LBs, membranes and cores. Extending this biochemical data, using structured illumination microscopy (SIM) to visualize LB-Core samples after virion fractionation we now show that RPL17 is found in LBs. This data is included as Fig. 3b in the revised version of the manuscript.

In addition, we have also added that "future assignment of ribosomal proteins as virion components should be verified using SR modalities" to the discussion section of the manuscript.

Reviewer #2: The mansucript has been improved and essential comments have been addressed by the authors.

We thank the reviewer.

Reviewer #3: 1. The first major problem with the study relates to the inadequate purification of mature virions. Unlike many other viruses, vaccinia virus is isolated from cell extracts rather than the cell culture supernatant, making purification from host materials more difficult.

The authors responded to my criticism of their purification in 3 ways - (1) citing

literature, (2) experimentally, and (3) by terminology.

With regard to (1), they justify their purification as “as field standard” but only in one of the half dozen or so papers cited was the purification for mass spectrometry and that was 16-years-old. The degree of purification needed depends on the proposed use and is very different for electron tomography and super resolution microscopy compared to mass spectrometry. Moreover, as mass spectrometry has improved and the depth of analysis increased, higher purity is needed. In a benchmark study not cited in the paper (Ngo, Mirzakhanayan and Gershon 2016) used two methods of purification, sucrose gradient and tartrate gradient, as the former separates by non-equilibrium rate zonal velocity and the latter by equilibrium buoyant density isopycnic banding. For each method hundreds of host proteins were detected by mass spectrometry. However, different host proteins were associated depending on the purification. By comparing the host proteins associated with mature virions by the two methods, the vast majority of cellular proteins were deemed non-packaged contaminants. Only ~60 overlapped by the two methods, and even these were still not excluded as contaminants. I did not notice any of the candidate LB host proteins of Bidgood et al in the that 60. In a related method, Resch et al. 2007 used two successive sucrose density gradient centrifugations followed by an isopycnic centrifugation and reported that many more host proteins were present prior to the isopycnic gradient (although the data were not shown).

Unfortunately, the landmark study by Ngo *et al.* is not a very useful guide for VACV purification as the materials and methods section “virus” does not provide a detailed protocol but refers to a previous manuscript that “briefly describes” purification via 36% sucrose cushion followed by 5-40% sucrose gradient and refers the reader to another previous manuscript, which is a book chapter that does not describe virus band-based purification.

Regarding the cell protein overlap, the two studies were done in different cell lines and to quote reviewer 4 “At some level, the concerns about impurities are the "nature of the beast". We have been cautious with our interpretation of the inclusion of cell proteins in LBs, and now caution readers to use additional imaging methods to confirm potential cell proteins components of LBs.

(2) One of the two methods used by the present investigators to establish purity is electron microscopy. Vaccinia virus is ~ 300 nm. While the EM might exclude intact mitochondria, which can be comparable in size, it does not exclude ribosomal subunits (note that one of the “candidate LB proteins is ribosomal), nor does it exclude small membrane fragments or sticky proteins. The second experiment was to show that some of the cell proteins detected by mass spectrometry were also detected by Western blotting. However, unlike the viral core protein, which was enriched between the 36% sucrose cushion and the sucrose gradient, the host proteins were severely diminished. While this experiment confirmed the mass spectrometry identification using antibodies, it does not provide additional data regarding their presence as contaminants or constituents.

We have now included structural illumination microscopy (SIM) images of ribosome protein RPL17 and mitochondrial protein Tomm20 that provide further evidence of their LB association. (Fig. 3b

(3) Simply inserting the word "candidate" before the host proteins is insufficient.

If the authors want to consider any of the host proteins as LB or even candidate LB, they need to further purify the mature virions by isopycnic gradient centrifugation. It is regrettable that the authors did not consider this during the planning stage of this study.

We disagree with the reviewer, they are candidate LB proteins *which require further assessment*, as indicated in the manuscript.

2. The second major problem with this study is the lack of any evidence that the LB proteins are modulators of the host oxidative antiviral response, and no additional evidence was provided in the revision. A critical first experiment would be to show that viral protein synthesis is not needed for the response, but this was not done. Secondly, the authors found that preventing expression of the candidate LB proteins did not affect the oxidative response and concluded that this is because of redundancy of LB proteins. The simpler explanation, not offered, is that none of those LB proteins are involved. In fact, no evidence was brought forward to support the conjecture that the LB redox or any other LB proteins affect the oxidative status of the cells although that is implicit in the title of the paper.

We have made our arguments clear in the last rebuttal, have used cautious language in the discussion and put forward our hypothesis based on the data and VACV multi-viral protein strategies for inhibition of other host defense mechanisms.

Reviewer #4: One good experiment would be to perform SIM/STORM microscopy to test/verify the localization of a few of the mitochondrial proteins.

We agree and have now added dual-color SIM images of RPL17 and Tomm20 to the manuscript. (Please see Figure 3b). We attempted to permeabilize virions with saponin to allow antibody access the LBs, but this was unsuccessful. To provide antibody access we removed the virion membrane using our fractionation protocol. SIM imaging showed that both RPL17 and Tomm20 (red) localize to LBs flanking the viral cores (green). As the virion fractionation protocol resulted in core expansion akin to what was seen by EM (Fig. S1), we could not apply VirusMapper to these images as alignment and segmentation relies on core asymmetry. On the plus side LBs were further apart on expanded cores allowing for visualization in both sagittal and frontal orientations.

Part III – Minor Issues: Editorial and Data Presentation Modifications
Please use this section for editorial suggestions as well as relatively minor modifications of existing data that would enhance clarity.

Reviewer #1: Line 109: Typo – "viral phosphatase H1L" (space and H missing in text)

corrected
Line 381 – You mean Fig S5a Yes
Line 387 – You mean Fig S5b Yes
Line 453 – If the data you are referring to is that presented in Fig S3, it doesn't actually show Hist1 to be differentially purified in infected vs uninfected cells. Fig 3a doesn't show differential purification between uninfected and infected cells. Please clarify what exact data you are referring to and be sure to comprehensively interpret the data (as per the above major comment).

Sorry for the confusion, we were referring to Fig 3a and have amended the sentence for clarity.

Line 458 – You don't use the possessive apostrophe for possessions of "it" – it's is an abbreviation of "it is". Either way, "of the VACV replicative niche" would be better.

We have modified the sentence

Reviewer #2: All minor issues raised previously have been addressed.

Reviewer #3: (No Response)
