# Supplementary material for: Poxviruses package viral redox proteins in lateral bodies and modulate the host oxidative response
Source: PLoS Pathog. 2022 Jul 14;18(7):e1010614. doi: 10.1371/journal.ppat.1010614 (PMC9282662; doi:10.1371/journal.ppat.1010614)
Supplement: Attachment — Submitted filename: 00_Bidgood_Response_to_Reviewers.docx [file ppat.1010614.s010.docx]

We thank the reviewers for their comments and suggestions. In line with these, we have now included additional experiments to address the major concerns raised (purity of virion preparations and link between redox proteins and VACV redox modulation). We have also modified the text (purification procedures) to be more accessible and to better reflect the findings.

These additions have served to strengthen our original findings, while consolidating and clarify our conclusions and experimental procedures as outlined in the point-by-point response below.

**Reviewer #1 (Evidence, reproducibility and clarity (Required)):**

Electron microscopic images of vaccinia virus (VACV) mature virions (MVs) reveal three main structures: an outer membrane, a core, and lateral bodies (LBs) between the core and membrane. It has been suggested that the components of the lateral body might interact with the host to prevent anti-viral defenses. Earlier studies from this laboratory used super resolution microscopy to localize three viral proteins to the LBs: a phosphoprotein, a phosphatase, and a glutaredoxin. The object of the first part of the paper was to identify additional proteins associated with the LBs. Having found some redox proteins, they investigated whether VACV modulates oxidative anti-viral responses upon infection.

**Major Comments**

1.The study begins with the characterization of viral and cellular proteins associated with fractions derived from purified VACV MVs. However, the authors fail to provide evidence of purity of the virus particles isolated from cell lysates, nor is the purity described in the reference given for the method used. The electron micrographs of core-LB and core fractions (Fig. S1) show a large amount of apparently adventitious material. Many of the viral proteins associated with these fractions, such as those belonging to the extracellular enveloped form of the virus, and the 586 cell proteins of which 210 were associated with the core and core-lateral body fractions, particularly those associated with mitochondria, could be from contaminating cellular material rather than incorporated into virus particles. However, neither the purity of the particles nor this interpretation is discussed and the abstract and text refer to these cell proteins as if they were bona fide core and LB-associated proteins. For this interpretation to be credible, strong evidence for purity of the virus particles and subfractions need to be provided.

We have utilized field standard VACV purification and virion fractionation methods which are well established and, with slight variation, used by the entire poxvirus community (Vaccinia virus and Poxvirology, Methods and Protocols, MIMB 269, chapter 8; and Yasuo Ichihashi, Masayasu Oie, and Takashi Tsuruhara. Location of DNA-Binding Proteins and Disulfide-Linked Proteins in Vaccinia Virus Structural Elements J Virol. 1984;50(3):929-938 and references therein). The purification procedure has been used in numerous EM, cryoEM, super-resolution imaging and mass spectrometry studies (Chung CS, Chen CH, Ho MY, Huang CY, Liao CL, Chang W. Vaccinia virus proteome: identification of proteins in vaccinia virus intracellular mature virion particles. J Virol. 2006;80(5):2127-40, Cyrklaff M et al. Cryo-electron tomography of vaccinia virus. PNAS. 2005; 102 (8) 2772-2777, -Gray RD, Beerli C, Pereira PM, Scherer KM, Samolej J, Bleck CK, et al. VirusMapper: open-source nanoscale mapping of viral architecture through super-resolution microscopy. Sci Rep. 2016;6:29132) to highlight a few.

To illustrate the purity of virus preps and for completeness, we have added EM images of the intact virions prior to fractionation (**new Fig. S1C; Whole VACV**). The whole virion purifications are free of cell debris, mitochondria, cell membranes, etc. This would dictate that the adventitious material, referred to by the reviewer, upon virion fractionation with NP40/DTT (**Fig. S1C; Pellet1 (LB-Core)**), and after additional trypsin treatment (**Fig. S1C; Pellet 2 (Core)**) are the viral (and cell) components dissociated from virions by the fractionation protocol.

To further illustrate the purity of virus preps we have now run uninfected and infected cell lysates through the VACV purification procedure. Immunoblot analysis for cellular proteins not detected in LBs (actin and tubulin) and those detected in LBs [Histone 1, RPL17 (ribosome), SOD1 and Tomm20 (mitochondria)] was performed after each step [cell lysis (nuclear pellet/supernatant), pellet from 36% sucrose cushion, virus band from 25-40% sucrose gradient, and final virus pellet].

In the uninfected cell purifications, actin, tubulin, histone 1, SOD1 and Tomm20 were left behind at the 36% sucrose cushion stage of purification, while RPL17 was carried through to the final pellet. In infected cell purifications, virus (A10) as concentrated enough for detection in the 36% sucrose cushion pellet. Cell proteins not found in LBs by mass spec (tubulin and actin) were lost during purification and not detected in the final virus pellet. With the exception of histone 1 the cell proteins detected in LBs by mass spec were also detected in the final virus pellet. Arguing for selectivity of packaging, less RPL17 and more SOD1 and Tomm20 were found in the final pellets of infected cell purifications. This data is now included as (**Fig. S3)** in the manuscript.

In conjunction with these experiments, and to address the reviewer’s point 3, we carried these purified virions through biochemical fractionation to qualify the localization of these cell proteins to LBs (**new** **Fig. 3A**). Purified virions were subjected to fractionation with 1% NP40/50mM DTT as described in the materials and methods and Figure S1. Fractionation was confirmed by immunoblotting for core (A10), membrane (A17) and LB (F17) components. Upon fractionation the membrane (A17) was separated from the Core-LB complex (A10 and F17). As expected, upon trypsinization of the Core-LB complex, the LB (F17) was lost while the core (A10) remained unaffected.

Regarding the cellular proteins, upon fractionation Histone 1 and Tomm20 remained exclusively with the LB-Core fraction, while RPL17 and SOD1 fractionated to both LB-Core and membrane. When the LB-Core complex was treated with trypsin each LB-Core associated cell protein was degraded to varying degrees: Histone 1 and Tomm20 were completely degraded suggesting their exclusive localization to LBs, the majority of RPL17 was degraded and a small amount of LB-Core associated SOD1 was found to be degraded, suggesting that these proteins are enriched in LBs, although they can be found in both LBs and Cores. These results serve as biochemical confirmation of the results of our mass spec experiment and confirm the validity of our fractionation procedure to define viral and cell LB candidate proteins.

Although this study was not designed to elucidate the association of cell proteins with the viral membrane, these additional experiments bring to light the possibility that a subset of cell proteins, akin to viral proteins (7 protein complex) A30 and G7 which fractionate with both VACV membranes and LB/Cores (Szajner P, Weisberg AS, Wolffe EJ, Moss B. J Virol. 2001 Jul;75(13):5752-61; Yasuo Ichihashi, Masayasu Oie, and Takashi Tsuruhara. J Virol. 1984;50(3):929-938). We have now alluded to this in the discussion

2.The identification of LB proteins depends on the removal of the membrane with a detergent, pelleting of the particulate material which is referred to as the core-LB fraction, followed by trypsin digestion and again pelleting the particulate material which is referred to as the core fraction. While trypsin digestion removes the lateral bodies, it does not mean that all proteins removed by trypsin are lateral body proteins. Therefore, the purification method is an enrichment and candidate LB proteins must be verified by other procedures, which was only done for 5 proteins

This is correct, the trypsin treatment enables identification through comparative enrichment of both LB proteins and proteins associated with the outer surface of the viral core. As a result, proteins that were already known to be *bone-fide* outer core and membrane proteins were discounted from the list of suggested LB candidate proteins.

To be more precise, throughout the manuscript we now refer to “LB proteins” as “**candidate** LB proteins” in cases where we have not verified LB residence by another method beyond the mass spec.

3.The abstract states that 15 viral LB proteins were identified. However, only five of the 15 were further analyzed by super resolution microscopy in this study so that the others should be referred to as candidate LB proteins. Similarly, the cell proteins should be referred to as candidate LB proteins as none of them were analyzed by super resolution microscopy even though 6 of them were reported to be more abundant than the most abundant viral protein.

As above we have adjusted the text to be more precise and have softened the language throughout. As discussed under point 1, we have confirmed the enrichment of 4 cellular LB candidates using biochemical fractionation (**new Fig. 3A**). In addition, we have also recently confirmed vimentin, identified by mass spec, as a LB candidate (Wood *et al.,* Cell Microbiol. 2021 Aug;23(8):e13334).

4.Fig. 4A shows a 3-fold reduction in ROS positive cells at 1 h after infection and much greater 23-fold reduction at 8 h. The higher inhibition at 8 h is attributed to post-replicative expression of LB proteins. However, by 8 h after infection, more than 100 viral proteins are expressed so why is this correlated with the LB proteins?

There are only 6 redox proteins encoded by VACV, 5 of which reside in LBs, E10 being the exception (see Schmidt et. al., Cell Rep. 2013 Aug 15;4(3):464-76). We see an impact on the % of ROS positive cells at 1h, long before late genes are expressed a much larger effect upon late expression (8 hpi). This is consistent with the early delivery and subsequent late expression of proposed LB-based ROS effectors.

**Minor comments**]

The last paragraph of Discussion and Abstract states that that all VACV-encoded redox proteins are in LBs. Are there other core or membrane proteins that have Cxx(x)C motifs? At least one, E10, a bona fide redox protein was not shown to be in LBs 

Thank you for pointing this out. This manuscript and our previous findings (Schmidt *et al.* Cell Reports 2013) indicate that E10 does not reside in the LBs. We have altered the wording of the manuscript to reflect this.

Reviewer #1 (Significance (Required)):

The most significant contribution of this study is evidence for the localization of four viral protein to LBs bringing the total number to 7. Additional candidate LB proteins were also suggested but not yet validated. While nearly 100 cellular proteins, some more abundant than the viral proteins, were also suggested to be LB proteins, contamination particularly with mitochondria was not ruled out. Interestingly, two of the LB proteins (A2.4 and G4) were known to be involved in the formation of disulfide bonds and additional ones have Cxx(x)C motifs suggesting that they may also be involved in redox. A small reduction in ROS positive cells was shown at 1 h after infection and a much larger reduction at 8 h; however, no evidence was presented to support the hypothesis that these reductions were mediated by LB proteins.

As outline in the point-by-point above, we have addressed the reviewers concerns regarding the purity of virus preps and cellular contamination. We have validated several cellular candidate LB proteins using biochemical methods and using a battery of mutant viruses linked the LB redox proteins to suppression of the host ROS response. We have also made additions to and adjusted the text in line with the reviewer’s comments.

**Reviewer #2 (Evidence, reproducibility and clarity (Required)):**

Bidgood and colleagues have use quantitative mass spectrometry to characterise the composition of VACV lateral bodies, proteinaceous structures analogous to the tegument of herpesviruses that have enigmatic composition and functions but are thought to deliver a bolus of protein that can modulate the host-cell environment before viral gene expression. They identify that VACV LBs contain redox-active viral proteins plus host-derived proteins. They go on to show that VACV infection reduces the abundance of reactive oxygen species in infected cells and that induction of oxidative stress is detrimental to virus replication. This work should serve as a useful reference for the poxvirus field, and adds to the body of literature confirming that redox state is yet another important host-cell environmental factor that viruses, and in particular large DNA viruses, seek to control during infection. 
Overall the paper is well written, the figures are clear, the experiments seem to have been performed in a technically robust manner and the description of the methods is sufficient to allow reproduction of their study.

There are a few major questions that arise:

1.While VACV replication is clearly more robust when the cell does not face oxidative stress (Fig. 4C), the redox capacity of LB-delivered redox-active proteins seems low (compare bottom panels of Fig. 4B). Do the authors think that there is a spatial aspect to the redox modulation by VACV LB-delivered proteins? This could be tested via microscopy by infecting cells expressing genetically-encoded redox sensitive reporters like Grx1-roGFP2. 

We thank the reviewer for this suggestion. We originally used a cytoplasmic/nuclear ROS reporter to capture the broad impact of VACV infection on the redox state of the cells. In line with your suggestion and as outlined above (Response to reviewer 1, point 4) we employed a cytoplasmic redox sensor, MitoSOX, to investigate role of the various LB redox proteins on the cell redox response to VACV infection. In line with the reviewer’s suggestion, we observed suppression of the cytoplasmic MitoSOX signal upon VACV infection suggesting there is a spatial aspect to VACV redox modulation.

To quantify the ability of mutant viruses to suppress a range of ROS activation we turned to flow cytometry as opposed to imaging. We obtained / generated a set of inducible and deletion mutants of the individual LB redox proteins. We utilized these to investigate the impact of LB redox protein loss on VACVs ability to suppress the host ROS response. We did not see an impact on VACVs ability to suppress host ROS responses upon individual knockout (**new Fig. 4 f)**. These results suggest that multiprotein knockout viruses are likely to be required to observe an effect. Unfortunately, these could not be generated and tested in the time frame of revision and will be the subject of future studies.

2.What is the relevance of the cellular proteins that co-fractionate with VACV LBs/cores? Many host-cell proteins or complexes are identified but the authors don't perform experiments to validate the presence of any of these proteins in LBs (e.g. using microscopy) and their (potential) functional relevance isn't addressed. The presence of so many mitochondrial and ribosomal proteins is particularly striking and raises concerns that mitochondria and ribosomes/polysomes may have co-sedimented with the VACV particles in the sucrose cushion experiment. If the authors have performed control experiments to discount this hypothesis they should be presented and, if not, this potential confounding factor should be noted.

We have addressed this point in the response to reviewer 1, point 1 above and the associated figures (**new Figs 3A, S1C and S3**). We refer the reviewer there for details. In short, we have performed control purification experiments and new fractionation experiments on a subset of candidate cellular LB proteins. The results indicate that 1-these proteins do not simply co-sediment with virions during purification 2-Provide biochemical confirmation of their enrichment in LBs vs Cores.

3.How relevant is the measurement of redox sensitivity in cultured cells to the context of VACV infection in vivo? Partial pressures of oxygen are higher in cell culture incubators than in animal issues, although rapid replication of immortalised cultured cells can lead to dramatic lowering of pericellular oxygen tension over the course of hours due to limited diffusion of O2 through culture medium. The authors should comment on the difference in oxidative stress experienced by cultured cells versus animal tissues in light of their results.

We thank the reviewer for pointing this out. Both the introduction and discussion review findings that overexpression of nitric oxide synthase (NOS2) the protein responsible for production of the major ROS species, superoxide, can restrict VACV *in vitro* and *in vivo* (Karupiah G, Xie QW, Buller RM, Nathan C, Duarte C, MacMicking JD. Science. 1993; 261(5127):1445-8)*.* In addition, NOS2 -/- mice are highly suspectable, as measured by lethality, to ectromelia virus (mousepox) infection compared to WT mice (Karupiah G, Chen JH, Nathan CF, Mahalingam S, MacMicking JD. Identification of nitric oxide synthase 2 as an innate resistance locus against ectromelia virus infection. J Virol. 1998;72(9):7703-6). These findings suggest that ROS is an important host defense mechanism against poxviruses.

4.The authors do not explain how VACV membrane proteins A26L and A6L are contained within VACV cores. Are these truncated soluble forms of the protein, for example lacking the transmembrane regions, or are they likely to associate tightly with the cores at the poles of the virion such that they remain associated after detergent removal of the membrane? The authors should comment.

Due to the highly interconnected nature of VACV particles we were not surprised to find MV membrane proteins that remained with the Core/LB complex after fractionation. Several manuscripts dedicated to investigating the molecular interactions within VACV have shown interactions between membrane, core and LB proteins even upon targeted degradation (Moussatche N, Condit RC. Fine structure of the vaccinia virion determined by controlled degradation and immunolocalization. Virology. 2015;475:204-18; Mirzakhanyan Y, Gershon P. The Vaccinia virion: Filling the gap between atomic and ultrastructure. PLoS Pathog. 2019;15(1):e1007508).

Figure S2 of the original manuscript incorporates these known literature-based interactions which largely explain the presence of the membrane proteins in our LB-enriched samples. The same would hold true for the core samples. We have added to the results section, that the inter-virion protein=protein interactions are likely to explain the presence of membrane protein contaminants.

5.The authors state that F17R constitutes 69% of LB mass (page 9 paragraph 2) but also that 6 human proteins were enriched and "at a greater abundance than the major LB viral protein F17R". F17R can't constitute more than 50% of the LB mass if there are other proteins more abundant in the LBs - can the authors please explain this discrepancy? 

We thank the author for pointing this out. This estimate of F17 LB mass arose from a volumetric estimate of VACV LBs based on EM and AFM images together with the reported copy number of F17 in virions and the specific partial volume of globular proteins (Schmidt et.al Cell reports, 2013). Any one or all 6 of the cellular proteins could be more abundant, but take up less LB mass based on their specific partial volume.

None-the-less, we agree that this is confusing and have removed reference to this throughout the manuscript.

**Minor Comments**

1.The authors state that they show hierarchical clustering of the proteomic results (Figs 1C, 2C) but the dendrograms of the clustering are not shown. These should be included. 

The dendrograms were removed for space and clarity. We have added them back to the figures.

2.In supplementary figure 1A the authors show quantitation of the lateral body versus core protein composition following variable trypsin treatment. However, they don't describe how this quantitation was performed, nor how many times it was attempted.

Thank you for pointing this out. We have added the method used for quantification of immunoblots to the Materials and Methods “Immunoblotting” section. This assay was performed multiple times using various ranges of trypsin. S1A is a single representative example showing a large range or trypsin concentrations to illustrate LB degradation and core stability. We have added this information to the Figure legend.

3.As described above for the viral cores, the authors should expressly comment on how they envisage that membrane proteins are identified in the LB proteome.

Please see the response to major point 4 above.

**Reviewer #3 (Evidence, reproducibility and clarity (Required)):**

The authors described the vaccinia virus lateral body (LB) proteome using quantitative comparative mass spectrometry. The majority of host proteins found were associated with mitochondria. In addition, 15 viral LB proteins were identified. Of the 15 viral LB proteins found, five are VACV redox proteins and their LB association was confirmed by state of the art microscopy (SIM-STORM and STORM-TEM). Finally, the authors demonstrate that vaccinia virus suppresses host cell oxidative responses, which may antagonize host antiviral responses. The data is presented in clear and comprehensive manner. Largely the conclusions are supported by the data. Appropriate numbers of experimental replicates have been performed.

**Major comments:**

1-Most conclusions are convincing. However, the link between the identified redox proteins and the anti-oxidative effect of VACV is not entirely clear. Could the authors express single VACV proteins and/or active site mutants of the viral redox modifiers to strengthen their conclusion that these LB proteins are causative of the observed redox status changes?

We thank the reviewer for this suggestion. We attempted to express the individual VACV proteins, under both natural and human codon optimized conditions, and the only protein that would express in the absence of infection was O2L. Therefore, we could not perform the suggested experiments.

In lieu of this we obtained / generated a set of inducible and deletion mutants of the individual LB redox proteins. We utilized these to investigate the impact of LB redox protein loss on VACVs ability to suppress the host ROS response. We did not see an impact on VACVs ability to suppress host ROS responses upon individual knockout (**new Fig. 4 f)**. These results suggest that multiprotein knockout viruses are likely to be required to observe an effect. Unfortunately, these could not be generated and tested in the time frame of revision and will be the subject of future studies.

2-The statement that poxvirus redox proteins are modulators of host oxidative anti-viral responses is not (yet) fully supported by the data presented. This should be addressed experimentally or alternatively the claim should be removed from the abstract, which would make the study a rather descriptive, but nonetheless informative, relevant and methodologically well executed study.

Please see response to point 1 above. We have adjusted the abstract and text throughout the manuscript to be more in line with the data. LB candidates are referred to as “candidate” proteins and redox proteins as “potential” modulators.

3-The statement that poxvirus redox proteins are modulators of host oxidative anti-viral responses is not (yet) supported by the data presented. If deletion mutant viruses cannot be generated, possibly expression of the single viral redox proteins could strengthen the claim. Recombinant protein expression and measurement of redox status of the cells seems feasible.

Please see response to point 1.

4-Data and the methods are presented in such a way that they can be reproduced.

We thank the review for their careful reading.

5-Experiments are largely adequately replicated and statistical analysis adequate. Statistical analysis for Fig. 4a is missing.

Statistical Analysis has been included for 4a and added to the figure and legend.

Proteomic analysis is performed according to current standards. Data deposition of the full proteomics dataset in a public database is highly recommended.

Mass spectrometry data is already available on the MassIVE repository and will be made publicly available upon publication.

**Minor comments**

•Prior studies are referenced appropriately. 

•Text and figures clear and accurate except for minor suggestions below.

Fig. 1C; Fig 2A. A hierarchical clustering may be preferable to the heat map.

For clarity, the dendrograms have been replace on the hierarchical clustering.

Fig 4. Statistical analysis should be performed to demonstrate that ROS production is significantly reduced by VACV.

Statistical Analysis has been included and added to the figure and legend.

Reviewer #3 (Significance (Required))

 The authors describe the first proteomic characterisation of poxvirus LBs, leading to the identification of four novel VACV LB proteins. Furthermore, VACV is shown to suppress cellular oxidative responses, which coincided with increased infection levels. This work builds on previous publications (Schmidt et al, 2013, Liu et al, 2014) and further characterises VACV LBs. Furthermore, the additional anti-oxidative role of the encased proteins is proposed. Virologists, especially those studying poxviruses and host-pathogen interactions should be interested in and influenced by the reported findings.

Field of expertise: Virology, proteomics.

We thank the review for this assessment

**Reviewer #4 (Evidence, reproducibility and clarity (Required)):**

**Summary:**

The work presented by Bidgood et al from the Mercer lab extends their prior studies of the poxvirus lateral bodies. In recent years, there has been an emerging hypothesis that these unusual virion features may serve a function akin to the herpesvirus tegument, delivering proteins upon infection that modukate host processes. Here, they successfully optimize a protocol to prepare subviral "cores" and "cores + lateral bodies" and use mass spectrometry to identify and compare the proteomes of each preparation. Superresolution microscopy is used to validate some of the spatial assignments. The most striking finding is the lateral body localization of the group of viral proteins known to function in the redox pathway and form disulfide-bonds within cytoplasmic viral proteins. They present further analyses suggesting that vaccinia "actively" suppresses oxidative stress (10%, rather than 40% of cells are ROS+), and that treatments that induce oxidative estress (TBHP) have a modest impact on levels of early and late transcripts and on viral yield.

The approaches are innovative and validated, the data are interesting and open up a new direction for research, and the paper is well written. However, there are some things that need addressed:

**Comments:**

1)Can the authors comment on the completeness of the mass spec analysis of the cores? L4, which is a major core component, isn't listed on the heat map/ H5, another abunndant protein, is listed - but it's hard to tell from the heat map why its abundance doesn't seem to change between the core and core + lateral bodies and doesn't seem to be abundant anywhere. Are other known core proteins missing?

We refer the reviewer to Table S1, Tab1. Of the 50 known “core” proteins (which are actually core and LB) (ref moss fields), we identified all except G5 which is also missing from all previous Mass Spec. This information has now been included in the text. As we were focusing on LBs, on the volcano and heat charts we set a cutoff for candidate LB proteins based on H1 enrichment. Because we are analyzing relative enrichment between LB-Core vs. Core samples, core proteins were anticipated to be present in both samples at equal amounts or to be enriched in the core sample after LB removal. L4 (VP8) falls into the equal amounts between samples ‘category’. As it does not make the H1 cutoff for enrichment, it’s represented as a grey dot on the volcano plot and not listed on the heat plot. Those core proteins whose abundance changed between LB-Core vs. Core samples (A10, A3 and H5 for example) are represented on the volcano and heat plots. In each of these cases the changes in abundance are minor and are likely due to the protein being more accessible to trypsin on the core surface (A10 and H5) during LB removal. We have added some additional information to the results section to clarify.

2)In their study of the redox proteins, they stress that the induction of ROS by TBHP decreases the level of early and late mRNAs as well as reducing viral yiield. However, even 100 uM TBHP decreases viral yield ~2-fold, and transcript levels less than that. These effects seem MUCH more moderate/minimal than the text suggests. The authors should adjust the text to be more in line with the data. Given the data, is it likely that this is why the redox proteins evolved and are delivered in the lateral bodies? Is there another possibility, or a more vulnerable cell type?

We have adjusted the text to be more moderate regarding the impact of TBHP on viral yield.

Despite the modest effects seen with TBHP on virus yield and lack of impact seen upon individual deletions, we are still in favor of a host modulatory role for the LB-redox proteins. We would argue that their importance as host modulators is evidence by 1-Their packaging in LBs and 2-functional redundancy.

1-Evolution deemed suppression of ROS important enough to deliver these 5 redox proteins into cells with incoming virus prior to the initiation of viral gene expression. In line with this, we see consistent. albeit modest, effects on early and late gene expression in the presence of TBHP (150µM and above), suggesting that early ROS production delays infection kinetics, which can impact virus replication, production, spread and likely immune detection.

2-While new data added to the manuscript (**Fig. 4E**) shows that viruses lacking single LB-redox proteins do not lessen VACVs ability to suppress ROS activation. This suggests there may be functional redundancy/cooperativity between the various redox proteins, which would make this akin the VACVs inhibition of NFκB, where multiple viral proteins are required for suppression.

Finally, it is important to point out that overexpression of nitric oxide synthase (NOS2) the protein responsible for production of the major ROS species, superoxide, has been shown to restrict VACV *in vitro* and *in vivo* (Karupiah G, Xie QW, Buller RM, Nathan C, Duarte C, MacMicking JD. Science. 1993; 261(5127):1445-8)*,* and NOS2 deficient mice are highly susceptible to ectromelia infection *in vivo* (Karupiah G, Chen JH, Nathan CF, Mahalingam S, MacMicking JD. Identification of nitric oxide synthase 2 as an innate resistance locus against ectromelia virus infection. J Virol. 1998;72(9):7703-6), suggests that ROS is an important host defense mechanism against poxviruses, this is covered in the introduction and discussion. It reasons that the poxvirus encoded LB-redox proteins might be important immunomodulators.

That ROS production by host cells is not limited to directly killing pathogens through oxidative damage. ROS directly and indirectly controls a multitude of signalling pathways that can trigger non-oxidative pathogen clearance, many of which may be overlooked in a tissue culture setting.

We tried multiple epithelial cell types, each of which gave similar results to those presented in the manuscript. That said, future experiments (not in the context of this manuscript) will be conducted with multi-deletion VACVs in macrophages where RNOS is thought to play a more significant role in pathogen control.

3)In describing the data in FIg 4 d-e, the authors conclude that the impact of TBHP is at the level of transcription, because transcript levels are moderately decreased. This conclusion is not justified - the impact could be at the level of transcript stability.

We thank the reviewer for pointing this out. We have modified the text to read that the impact could be at the level of transcription or transcript stability.

4)In their introduction, the authors state conclusively that vaccinia virus enters cells by macropinocytosis. Although there is good evidence for this mode of entry (from the senior author of this work during an earlier career stage), there are other investigators who have reported that entry can also occur via direct fusion at the plasma membrane. The authors should modify their statement.

We have modified the statement and included the associated references.

Reviewer #4 (Significance (Required)):

The work is well done and significant, and the authors have advance dour understanding of poxviral lateral bodies. The combination of different technical approaches is superb.

The encapsidation of the viral redox proteins into the cytoplasm upon entry is intriguing. Despite the rather modest impact that the authors show upon inducing oxidative stress, the hypothesis that vaccinia actively combats ROS immediately upon infection is provocative and worth studying.

The work will be of interest to a broad audience of virologists.

We thank the reviewer for these comments
